# Structure of Rhoptry Neck Protein 2 is essential for the interaction in vitro with Apical Membrane Antigen 1 in *Plasmodium vivax*

**DOI:** 10.1186/s12936-019-2649-6

**Published:** 2019-01-25

**Authors:** Perla Salgado-Mejias, Flavio L. Alves, Kátia S. Françoso, Karin A. Riske, Emerson R. Silva, Antonio Miranda, Irene S. Soares

**Affiliations:** 1 Department of Clinical and Toxicological Analyses, School of Pharmaceutical Sciences, University of São Paulo, São Paulo, SP Brazil; 2 Departamento de Biofísica, Universidade Federal de São Paulo, São Paulo, SP Brazil; 3 Department of Chemical Sciences and Natural Resources, Faculty of Engineering and Science, University of La Frontera, Temuco, Chile

**Keywords:** *Plasmodium vivax*, PvAMA1, PvRON2, Protein–protein interaction, Peptide–protein interaction

## Abstract

**Background:**

In several *Apicomplexa,* the formation of moving junctions (MJs) at the interface between the external membranes of the invading parasite and the host cell is essential for the process of parasite invasion. In *Plasmodium falciparum* and *Toxoplasma gondii*, the MJ is composed of the Apical Membrane Antigen 1 (AMA1) and Rhoptry Neck Proteins (RONs) complex; specifically, AMA1 interacts with RON2 during host cell invasion.

**Methods:**

Recombinant proteins based on *Plasmodium vivax* RON2 (A2033-P2100) and its synthetic peptide fragments, one cyclic and one linear, based on PvRON2 (D2035-T2074) were generated and used to evaluate the interaction with *P. vivax* AMA1 (PvAMA1) by the far western blot, surface plasmon resonance (SPR), and isothermal titration microcalorimetry (ITC) methods. The structural studies of peptides were performed by circular dichroism, and the structural analysis of the complex of PvAMA1 with peptides based on PvRON2 (D2035-T2074) was conducted with small-angle X-ray scattering (SAXS).

**Results:**

Surface plasmon resonance (KD = 23.91 ± 2.078 μmol/L) and ITC (K = 3 × 10^5^ mol/L) studies conclusively showed an interaction between the cyclic peptide based on PvRON2 and PvAMA1-His_6_. In contrast, the linear peptide and recombinant PvRON2 (GST fusion protein) did not show an interaction with PvAMA1. However, the interaction among recombinant proteins PvRON2.2 and PvAMA1-His_6_ was possible to show by far western blot.

**Conclusions:**

The results show that the PvRON2 structure, particularly the S–S bond between C2051 and C2063, is determinant for the existence of the interaction between PvAMA1 and PvRON2.

**Electronic supplementary material:**

The online version of this article (10.1186/s12936-019-2649-6) contains supplementary material, which is available to authorized users.

## Background

The fight against malaria is considered one of the priority areas for research and vaccine development [1]. Attempts to develop malaria vaccines are primarily focused on *Plasmodium falciparum* and are directed towards reducing morbidity and mortality [2]. Although *Plasmodium vivax* is globally the most widely distributed and the most prevalent species in America [3], clinical trials performed with *P. vivax* vaccine candidates have to date only advanced to phase I with three antigens [4].

Aiming to develop vaccines against this species, in recent years, the group studied multiple aspects of naturally acquired immune responses against recombinant proteins representing *P. vivax* blood-stage antigens. Among the most promising malaria vaccine candidates, it has been found that the recombinant proteins representing Apical Membrane Antigen 1 (AMA1) were highly immunogenic in natural infections [5–9] and after mouse immunization [9–11]. Using homologous and heterologous prime-boost protocols with recombinant protein and/or adenovirus based on AMA1, different vaccination protocols have been established in mice [9, 11].

Despite the use of AMA1 for vaccine development, little is known about the function of this protein or the antibodies induced by natural infection or immunization with recombinant proteins. In the specific case of *P. vivax*, the absence of efficient and continuous in vitro culture systems has imposed limitations on in vitro testing of potential vaccine candidates [12]. Moreover, protection studies are complex because this species does not infect rodents. The functional importance of PvAMA1 in invasion is derived from a study in which antibodies induced by immunization with recombinant PvAMA1 were able to partially inhibit the red blood cell re-invasion ex vivo [9].

The formation of a moving junction (MJ) at the interface between the external membranes of the invading parasite and the host cell is apparently a common feature across the Apicomplexa phylum [13]. Recent studies with *Toxoplasma gondii* and *P. falciparum* showed that AMA1, which is secreted from micronemes, interacts with Rhoptry Neck Protein 2 (RON2) during the host cell invasion [14], and the sequence and structural data from the AMA1 and RON2 proteins suggest that their interaction is highly conserved across the Apicomplexa phylum [15]. Therefore, it has been hypothesized that this complex is conserved in *P. vivax,* and this AMA1–RON2 interaction offers potential for the development of anti-infective (vaccines and/or drugs) strategies. In fact, during the development of the present study, two other works showed the interaction of peptides and recombinant proteins based on PvRON2 and PvAMA1 [15, 16].

In 2017, Vulliez Le Normand et al. [15] showed that a 39-residue peptide based on PvRON2 presented cross-reactivity between AMA1 of *P. vivax* and *P. falciparum*. Their results showed that between *P. falciparum* and *P. vivax*, some intermolecular contacts are common to the AMA1/RON2 pair, while others are particular to certain species; the RON2 ligand adapts to sequence differences in the AMA1 binding groove. For this reason, PvAMA1-DII-III is able to bind to both PvAMA1 and PfAMA1 in spite of some differences in the intermolecular interactions. On the other hand, Bermudez et al. [16] showed a stronger interaction of PvRON2 with PvAMA1-DII-III than with the PvAMA1 ectodomain or PvAMA1-DI-II, suggesting that other PvAMA1 regions may participate in the interaction with PvRON2. Furthermore, they also demonstrated that another PvRON2 functional region located towards the central region participates in the interaction with reticulocytes and interacts strongly with PvAMA1.

In *P. falciparum* and *Toxoplasma gondii,* several studies showed that antibodies or peptides that prevent formation of the AMA1–RON2 complex also block invasion [15, 17–22]. Once the AMA1–RON2 complex formation in *P. vivax* is confirmed, this assay can be explored to evaluate the functionality of the antibodies generated by immunization with recombinant *P. vivax* AMA1 and/or RON2. In the present study, it has been evaluated the binding of the RON2 peptides to the AMA1 protein of *P. vivax*.

## Methods

### Synthesis, cloning, yeast expression and purification of *Pv*AMA1-His_6_

The recombinant PvAMA1-His_6_ was obtained as previously described by Vicentin et al. [9].

### Synthesis, cloning, bacterial expression and purification of PvRON2 recombinant proteins

In the amino acid sequence of the *P. vivax* RON2 protein (PVX_117880) [23], which was obtained from the base pairs of the gene that encodes this protein, we focused the region between residues 2033 to 2100, which corresponds to the sequence of the fusion proteins GST-RON2.2 and GST-RON2.2 mut (C2051A and C2063A). Recombinant *E. coli* Bl21 bacteria were transformed with the PGEX-3x vector (GE Healthcare—Chicago, IL) which contains the gene for expressing Glutathione-S-Transferase (GST) (26 kDa), GST-RON2.2 (33 kDa) or mutant GST-RON2.2mut (33 kDa). The pre-inoculations were performed with a colony of each bacterium that were incubated in 20 mL of Luria Broth (LB) medium supplemented with ampicillin (100 μg/mL) (Sigma—San Luis, MO) at 37 °C under agitation overnight. Then, this pre-inoculum was transferred to 180 mL of culture medium supplemented with ampicillin (100 μg/mL) at 37 °C under agitation until it reached an OD of 0.6–0.8. After this step, the cultures were supplemented with 0.1 mmol/L IPTG and incubated under the conditions described above for 5 h. Then, the culture was centrifuged at 4000 rpm for 15 min at 4 °C, and the culture supernatant was treated with PBS/1% Triton, 4 mg/mL of lysozyme, and PMSF (1.0 mmol/L) and lysed by sonication for 40 min. After bacterial lysis, the supernatant was filtered with 0.45 μm filters and subjected to purification of the proteins in the supernatant.

Proteins were purified by fast protein liquid chromatography (FPLC) in two stages: (i) the affinity stage in 1 mL GSTrap FF glutathione resin (GE Healthcare), using a buffer composed of 50 mmol/L Tris–HCl, 10 mmol/L reduced glutathione; and (ii) the ion exchange chromatography stage with a compound buffer of 20 mmol/L Tris–HCl/1 mol/L NaCl pH = 8.0, in Q-Sepharose resin (GE Healthcare), coupled to a ÄKTA™ prime liquid chromatographer (GE Healthcare). The obtaining purified protein was checked by 12% SDS-PAGE gel electrophoresis.

### Far western blot

For these experiments the strategy used membrane-based Far WB without denaturing/renaturing, in which the proteins are subjected to electrophoresis under denaturing conditions, and then transferred to the membrane for incubation with the purified bait recombinants protein(s) [24]. The PvAMA1-His_6_, GST-PvRON2.2, GST-PvRON2.2 mut (C2051A and C2063A), and GST (separately) proteins were run on 12% SDS-PAGE gels using 140 V for 70 min at room temperature. After electrophoresis was complete, the proteins were transferred to nitrocellulose membranes (GE Healthcare) using 100 V for 1 h at 4 °C. The nitrocellulose membranes were incubated with blocking solution composed of 5% skim milk and 2.5% bovine serum albumin (SIGMA) in PBS for 3 h at room temperature. Subsequently, the membranes were incubated without previous washes with PvAMA1-His_6_ at 40 μg/mL prepared in blocking solution overnight at 4 °C. After three washes of 10 min each, the nitrocellulose membranes were incubated with the anti-DII monoclonal antibody PvAMA1 (1: 1000) and the anti-GST polyclonal antibody (1: 500) in blocking solution for 1 h at room temperature. After three washes as mentioned above, the nitrocellulose membrane were incubated with goat anti-mouse IgG secondary antibody conjugated to peroxidase (Seracare, Milford, MA) (1: 1000) in blocking solution for 1 h at room temperature. After washing, the development was carried out for 15–45 s using the GE Healthcare ECL Plus Western Blotting Detection System kit.

### *Pv*RON2-based peptide synthesis and purification

The PvRON2 (2035–2074) linear peptide was purchased from Peptide 2.0 (Chantilly, VA) with a level of purity higher 95%. The synthesis of the PvRON2 (2035–2074) cyclic (Additional file 1) peptide was done manually according to the standard protocols on a Fmoc-Thr(tBu)-Wang Resin (0.624 mmol/g) in a 1.5 mmol scale. Unless otherwise stated, Fmoc was removed with 20% piperidine/DMF for 20 min, followed by DMF washes. Couplings and recouplings were performed with a mixture of 5.0 eq. of Fmoc-amino acid in the presence of DIC/HOBt (1:1) in DCM/DMF (1:1) for 120 min. Peptide cleavage and deprotection were performed by using the K reagent cleavage cocktail for 6 h. The crude peptides were separated from water insoluble non-peptide material with 5% acetic acid in water. The resulting peptide solution was kept at pH 6.8–7.0 and 5 °C for 72 h. The formation of disulfide bridges between C2051 and C2063 was monitored by reversed-phase liquid chromatography coupled to electrospray ionization mass spectrometry (LC/ESI–MS).

All reagents and solvents for solid-phase peptide synthesis were of analytical grade and were used from freshly opened containers without further purification. Resins were purchased from AAPPTec (Louisville, KY), and the MBHA resin was from Advanced ChemTech (Louisville, KY). The protected amino acids were purchased from Bachem (Torrance, CA) with the following side chain protections: Asn(Trt), Asp(OtBu), Cys(Trt), Glu(OtBu), Ser(tBu), Thr(tBu), and Tyr(tBu) for the Fmoc strategy.

The purification of cyclic PvRON2 (2035-2074) was carried out on a Waters 600 HPLC instrument using a Jupiter C_18_ semi-preparative column (21.2 × 250 mm, 300 Å pore size, 15 µm particle size, Phenomenex—Torrance, CA). This process was performed in two steps: (1) Solvent A: 0.05% HFBA in water; and Solvent B: 60% ACN in 0.05% HFBA. The linear gradient was 50–80% B for 180 min with a flow rate of 10 mL/min and detection at 220 nm; (2) Solvent A: 0.1% TFA in water; and Solvent B: 60% ACN in 0.1% TFA in water. The linear gradient was 40–70%B for 90 min with a flow rate of 10 mL/min and detection at 220 nm. For purification, cyclin PvRON2 (2035–2074) was dissolved in 0.05% HFBA or in 0.1% TFA. The fractions were screened under liquid chromatography coupled to a mass spectrometer (LC/ESI–MS), and fractions of satisfactory purity were pooled and lyophilized.

For LC/ESI–MS, data were obtained on a Waters instrument (model 3100 (Milford, MA) coupled to a Waters Alliance system (model 2695) using a Phenomenex Gemini C18 column (2.0 mm × 150 mm, 3.0-μm particle size, and 110-Å pore size). Solvent A was 0.1% TFA in water, and solvent B was 60% ACN in solvent A. The gradient was 5–95% B for 30 min with a flow rate of 0.250 mL/min, and peptides were detected at 220 nm. Mass measurements were performed in a positive mode with the mass range between 200 and 2000 m/z.

### Circular dichroism

Circular dichroism (CD) spectra were recorded in a Jasco 720 spectropolarimeter (Oklahoma, OK) using a cylindrical quartz cell of 1 mm pathlength with a 1 nm bandwidth, 0.2 nm resolution, 4 s response time and a scan speed of 50 nm/min. All CD spectra were recorded after accumulation of 8 runs and smoothed using an FFT (Fast Fourier Transform) filter to minimize background effects. Spectral baselines were corrected by solvent subtraction. Data are expressed as molar ellipticity, [*Ɵ*] (10^3^ × deg × cm^2^/dmol). Peptides samples were solubilized in water and H_2_O/TFE (1:1).

### Binding studies by surface plasmon resonance (SPR)

Surface plasmon resonance measurements were made in a Biacore T200 instrument (GE Healthcare). PvAMA1-His_6_ protein diluted in 10 mmol/L sodium acetate pH 4.0 was covalently immobilized by an amine-coupling procedure on a CM5 sensor chip (GE Healthcare). The reference flow cell was prepared by the same procedure in the absence of protein. Bindings assays were performed at 25 °C in PBS by injecting a series of peptide PvRON2 (2035–2074) (cyclic and linear) at different concentrations at a constant flow rate of 50 µL/min. Peptide dissociation was realized by injecting the running buffer, and the surface was regenerated by injecting glycine/HCl pH 2.0 followed by NaCl 2 mol/L pH 7.2. Control flow cell sensorgrams were subtracted from the ligand flow cell sensorgrams, and averaged buffer injections were subtracted from the analyte sensorgrams. All calculations were made using the Biacore T200 Evaluation Software, version 1.0, and figures were made using GraphPad Prism 5.0. The saturation curves were obtained by plotting Req versus the peptide concentration that was fitted with a steady-state model to obtain the apparent equilibrium dissociation constant, KD.

#### Binding studies by isothermal titration microcalorimetry (ITC)

ITC measurements were made using a MicroCal VP-ITC calorimeter (Malvern Instruments—Malvern, Worcestershire). PvAMA1-His_6_ and cyclic and linear PvRON2 (2035–2074) peptides were diluted in PBS to final concentrations of 9.43 µmol/L, and 300 µmol/L, respectively. PvAMA1-His_6_ (initial volume 2.5 mL) was titrated at 25 °C by consecutive injections of the peptides (10 µL aliquots at 10 min intervals). Raw data were normalized and corrected for the heat of the dilution of peptides in PBS. The binding stoichiometry was determined by fitting the final data to a one-site interaction model using the Origin 8 software (OriginLab—Northampton, MA).

#### Structural studies by small-angle X-ray scattering (SAXS)

SAXS experiments were carried out on the SAXS-1 beamline at the Brazilian Synchrotron Light Laboratory (LNLS, Campinas). X-rays energy was 8 keV (l = 1.542 Å). Data collection was performed by a Pilatus 300 K CCD detector and approximately 300 µL of aqueous solutions containing protein and peptides were injected in between the mica windows with a path length of 1 mm. Ten frames, 30 s each, were collected and, if no radiation damage was detected, they were averaged, and the background was subtracted. Data processing was carried out by using the indirect Fourier transform (IFT) method [24] in order the pair-distance distribution functions (P(r)) in the GNOM program [25]. In the following, the obtained P(r) function was inputted in the DAMMIF program [26] to perform ab initio reconstructions and provide low-resolution models of the particles in solution.

## Results and discussion

### Structures of PvRON2-based peptides

**Fig. 1:**
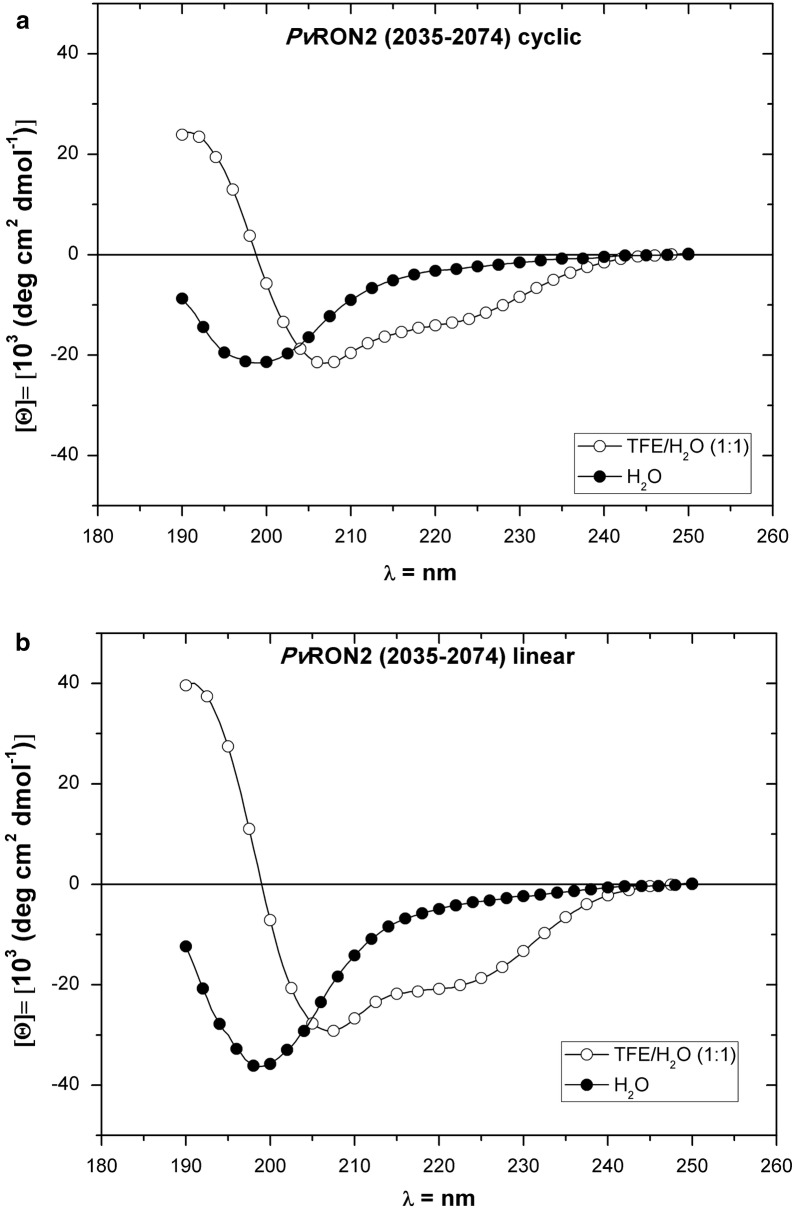
CD spectra in H_2_O of PvRON2-based peptides in the presence of H_2_O and TFE/H_2_O (1:1). The conformational properties of PvRON2-based peptides were examined by CD in an aqueous solution containing the structure-inducing solvent TFE. In water, both peptides presented a mixture of PPII and random coil conformations. **a** For the cyclic peptide, this conformation changed in the presence of TFE/H_2_O (1:1) to a 3_10_ helix conformation with α-helices contributions. **b** For the linear peptide, this conformation changed in the presence of TFE/H_2_O (1:1) to a predominantly α-helical conformation

The conformational properties of peptides based on PvRON2 (2035–2074) were examined by CD in water and aqueous solutions containing the structure-inducing solvent TFE. The objective of these experiments was to answer questions related to the conformational behaviour of the peptide in a water/membrane transition. Figure 1 shows CD spectra of peptides PvRON2 (2035–2074) cyclic and linear. The experiments in water demonstrate the prevalence of mixture of Polyproline II (PPII) with random coil conformation for both peptides. For cyclic PvRON2 (2035–2074), the spectra profiles suggest a 3_10_ helix conformation with contributions from α-helices in TFE/water. For the linear peptide, the CD spectra suggest a higher presence of α-helical conformation with lower contributions of other structures, like the 3_10_ helix in TFE/water.

The comparatively lower α-helical content observed for the cyclic peptide of PvRON2 (2035–2074) could be due the disulfide bond between C2051 and C2063; disulfide bridge cause a great conformational restriction to the cyclic peptide, which is reflected in its CD spectra profile. The results for cyclic PvRON2 (2035–2074) are not in agreement with those obtained by Vulliez-Le Normand et al. [15]; they reported an N-terminal segment that begins with a short α-helix from residues S2037 to I2043, followed by an extended peptide stretch to a disulfide-linked β-hairpin formed between C2051 and C2063, and a C-terminal region following C2063 that is extended but with no regular structural elements. This difference in results may be due to the different conditions in which the studies were conducted.

These results indicate that the predominant structure of the region between residues D2035-T2074 in the peptides studied in this work is PP II in aqueous medium. The polyproline left‐handed helical structure was often confused with unordered, disordered, irregular, unstructured, extended, or random coil conformations. The only way to unambiguously reveal PPII structures in solution is to use spectroscopies based on optical activity, such as circular dichroism (CD), vibrational circular dichroism (VCD), and Raman optical activity (ROA), since this structure is difficult to detected by X-ray crystallography or NMR spectroscopy. However, the identification of the PPII structure by CD is widely considered to be the most reliable methodology [27]. This structure is essential to biological activities such as signal transduction, transcription, cell motility, immune response and molecular recognition [28]. For the cyclic peptide in TFE/water, the CD spectra suggest the presence of a 3_10_ helix structure; this structure is frequently found in transmembrane domains of membrane proteins [29], and this structure is a transition structure between a random coil and an α-helix in short peptides [30, 31].

### Binding studies between peptides (PvRON2) and recombinant proteins based on PvRON2 and PvAMA1

**Fig. 2:**
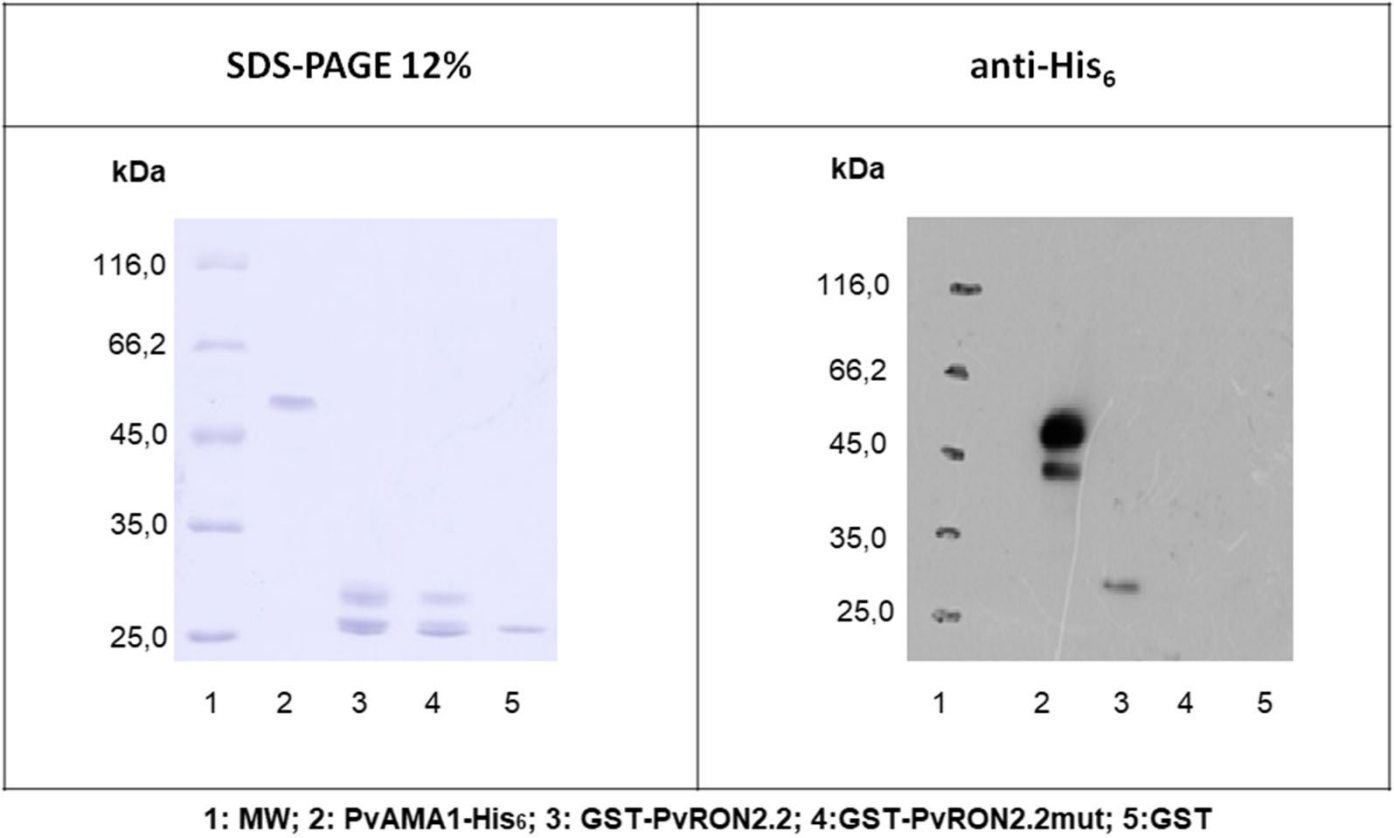
Far western blot of PvAMA1-His_6_ and recombinants proteins based on PvRON2. Far western blots performed after 12% SDS-PAGE electrophoresis of the PvAMA1-His_6_, GST-PvRON2.2, GST-PvRON2.2 mut and GST proteins. The membranes were incubated with PvAMA1-His_6_. Western blots were performed with the anti-DII antibody against PvAMA1-His_6_. A marking corresponding to PvAMA1-His_6_ can be seen in the position of GST-PvRON2.2, and this does not occur in the other positions. The experiment was repeated three times

Initially, the far western blot was used to evaluate the interaction between the recombinant protein PvAMA1-His_6_ and proteins based on PvRON2. PvAMA1-His_6_ was incubated with a membrane on which GST-PvRON2.2, GST-PvRON2.2 mut, and GST were immobilized. The samples shown in the SDS-PAGE are the same used in the anti-His_6_ panel (Fig. 2). A proportion of the recombinants proteins based in RON2.2 might have suffered degradation which has resulted in a smaller band (~ 25 kDa) representing GST. Considering PvAMA-1 (lane 2), western-blotting is much more sensitive than SDS-PAGE stained with Coomassie which might have revealed this second band that has been observed and reported previously [9]. This result shows the presence of an identification marker for PvAMA1-His_6_ protein in the position corresponding to the immobilized GST-PvRON2.2 protein (Fig. 2), which indicates the existence of an interaction between PvAMA1-His_6_ and GST-PvRON2.2. This interaction does not occur between PvAMA1-His_6_ and GST-PvRON2.2 mut which may suggest that the disulfide bond, and consequently, the protein conformation is important for the interaction between PvAMA1 and PvRON2, which is consistent with the results published by Tonkin et al. for *Toxoplasma gondii* [18]. GST does not prevent this interaction, but GST in recombinant proteins generates strong steric hindrance. Therefore, it was decided to use synthetic peptides in the following experiments.

**Fig. 3:**
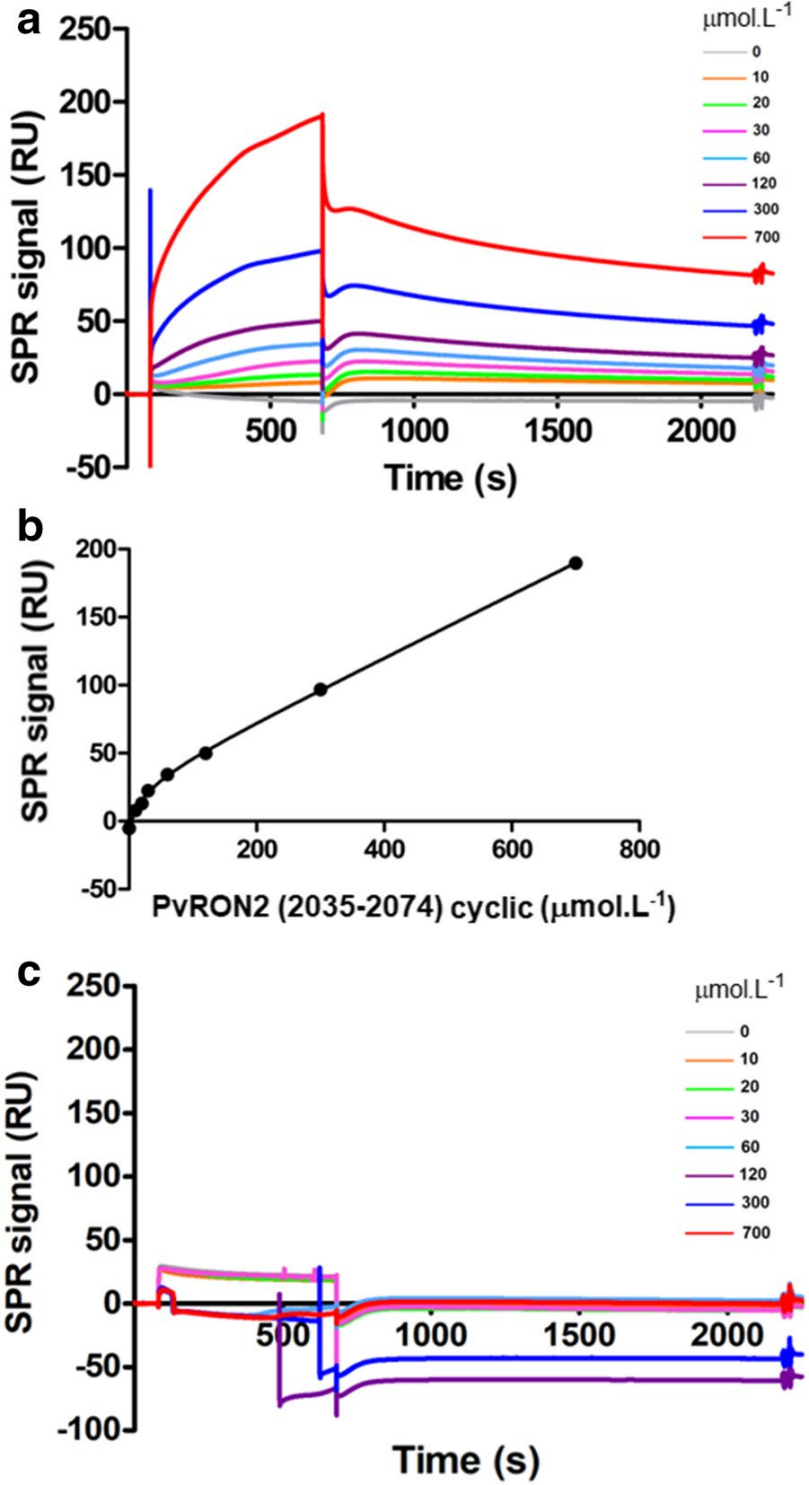
Surface plasmon resonance studies of peptides based on PvRON2 (2035–2074) binding to PvAMA1-His_6_. **a** Sensorgrams showing the cyclic PvRON2 (2035–2074) peptide binding to PvAMA1-His_6_. The concentrations of the cyclic PvRON2 (2035–2074) peptide are indicated for each curve (µmol/L). **b** The kinetics of the interaction between cyclic PvRON2 (2035–2074) and PvAMA1-His_6._ KD is 23.91 ± 2.078 µmol/L. **c** Sensorgrams showing that linear *Pv*RON2 (2035–2074) does not interact with PvAMA1-His_6_. The linear PvRON2 (2035–2074) concentrations are indicated for each curve (µmol/L)

The interaction between PvAMA1-His_6_ and PvRON2 was confirmed by SPR and ITC experiments employing the synthetic peptides. The SPR results show the interaction between PvAMA1-His_6_ and the cyclic PvRON2 (2035–2074) peptide (KD = 23.91 ± 2.078 μmol/L) (Fig. 3a, b) but no interaction between PvAMA1-His_6_ and the linear PvRON2 (2035–2074) peptide (Fig. 3c). This found confirms that the structure formed and stabilized by the disulfide bond between C2051 and C2063 in cyclic PvRON2 (2035–2074) is essential for this interaction, as was suggested with the results of far western blot. The result obtained for the cyclic peptide was different than previously reported by Vulliez Le Normand et al. [15], however, to analyse these results it is important to highlight that the purity level of the peptides used in all experiments of this work were higher than 95%, (Additional file 1) that is required for reliable SPR experiments [32]. This result in agreement with the report from Bermudez et al. [16] for a recombinant protein that contain a PvRON2 sequence in which the peptides studied were based.

**Fig. 4:**
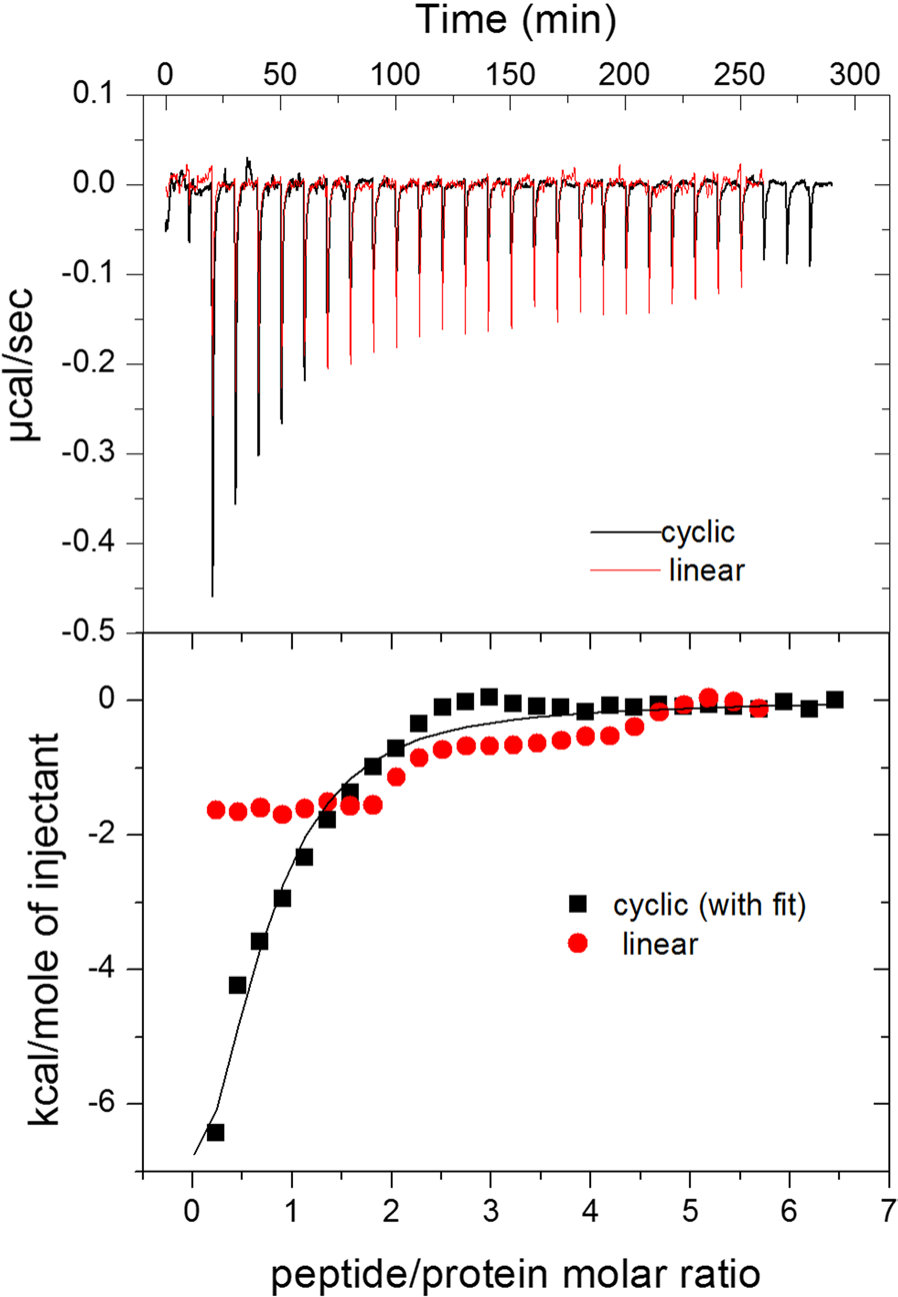
Isothermal titration calorimetry studies of the cyclic (2035–2074) and linear PvRON2 peptides binding to PvAMA1-His_6_. Heat flow (upper panel) and integrated heat per injection (lower panel) along the titration of 9.43 µmol/L PvAMA1-His_6_ with the peptides (cyclic and linear) (10 µL injections of 300 µmol/L peptide). The heat of dilution was subtracted from the data. The line in the lower panel is a fit using the one-site model (ΔH = − 36 kcal/mol, K = 3 × 10^5^ M^−1^, n = 0.6)

In the ITC studies (Fig. 4), thermograms obtained from the injection of cyclic and linear PvRON2 (2035–2074) into a solution of PvAMA1-His_6_ show exothermic peaks. The interaction is stronger with the cyclic peptide, as can be seen from the integrated heats in the lower panel. A fit using the one-site model revealed an interaction of the cyclic peptide with PvAMA1-His_6_ with a stoichiometry of 0.6 peptide/protein and a relatively high affinity (K = 3 × 10^5^ M^−1^). These results of affinity by ITC are concordant with the results of SPR obtained above in this work.

### Structural studies by SAXS

Synchrotron SAXS experiments failed to determine the molecular structure of the PvAMA1/PvRON2 complex under the experimental conditions with cyclic peptide because to the low amount of material due to the synthesized cyclic peptide based on PvRON2 had high purity but low synthesis yield (Additional file 1). This low yield may be due to difficulty in cyclizing the peptide due to the three prolines (P2057, P2058, P2059) found in the centre of the loop formed by the S–S bond between C2051 and C2063. For this reason, all cyclic peptide was used in CD and interaction experiments to make the comparison with the results obtained with the linear peptide.

**Fig. 5:**
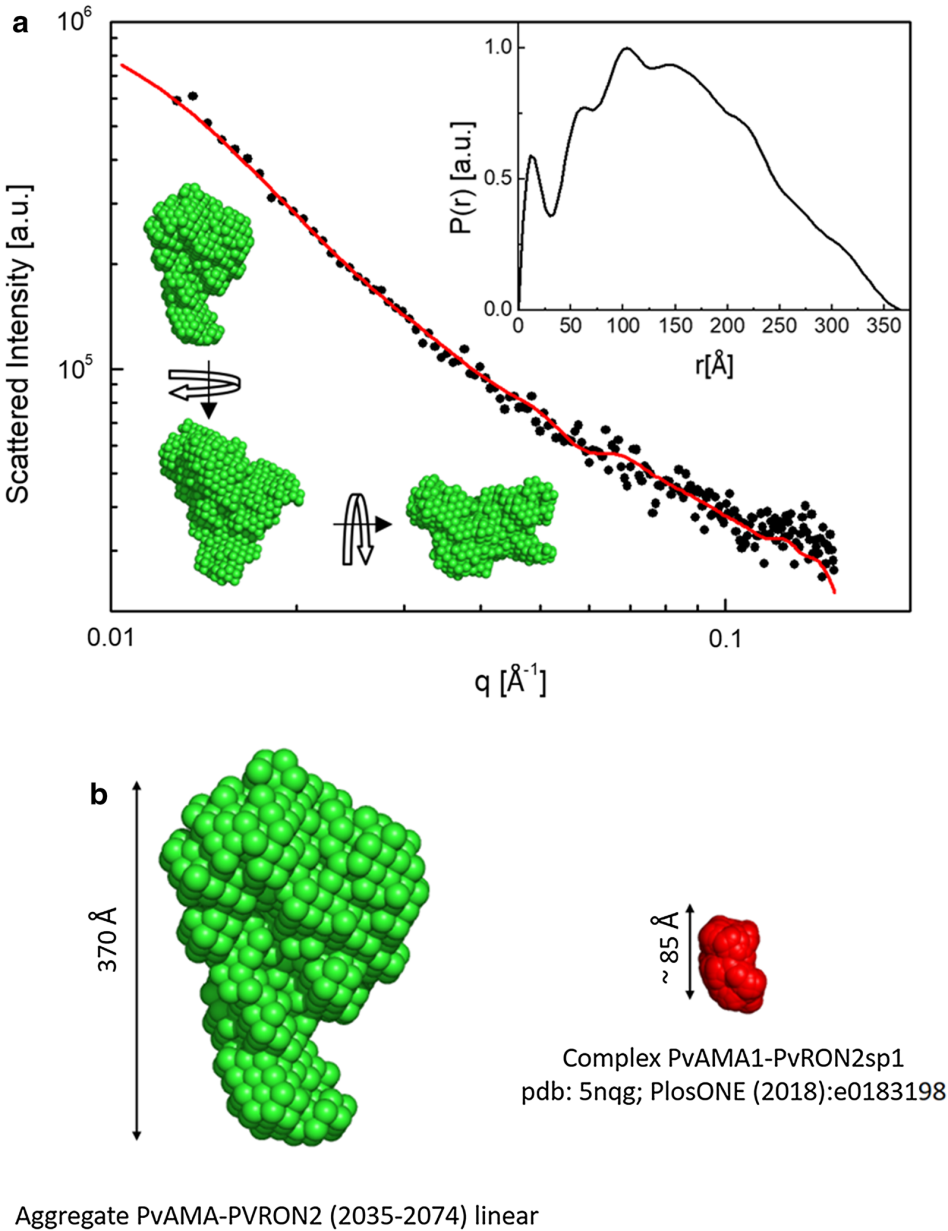
Small-angle X-ray scattering studies of the linear PvRON2 (2035–2074) peptide and PvAMA1-His_6_. SAXS data from solution containing the recombinant PvAMA1-His_6_ protein and linear PvRON2 (2035–2074) peptide. The results show the formation of aggregates under the experimental conditions used in the study. **a** The red line corresponds to the best model fitting the data (χ^2^ = 2.57). Insets: P(r) function obtained through indirect Fourier transform shows the presence of aggregates with maximum sizes close to 370 Å. **b** The average 3D model reconstructed from the P(r) function indicates aggregates with irregular shape. The aggregates in solution have dimensions about four times bigger than the complex between PvAMA1-PvRON2sp1 previously reported by Vulliez-Le Normand et al. [15]

The experiments carried out between PvAMA1-His_6_ and linear PvRON2 (2035–2074) showed that no complex was formed between these two molecules. However, under these experimental conditions [PvAMA1-His_6_ 0.943 μmol/L and PvRON2 (2035–2074) linear 300 μmol/L], it was observed the formation of aggregates much bigger than that reported in the literature for the Apicomplexa AMA1/RON2 complex [15, 22, 33] (Fig. 5). SAXS data were properly fitted using indirect Fourier transform [31] to obtain the corresponding P(r) function of the objects in solution. This function represents the distribution of distances between pairs of points in scattering objects in solution and from its profile it is possible to devise quantitative information on size, gyration radius and average shape of the particles. By using the P(r) function (inset in Fig. 5), low-resolution models of the aggregates were obtained through ab initio approaches [26] which showed the presence of particles with irregular shape in solution. The maximum size found in these objects was 370 Å, whereas the gyration radius was found at 120 Å. Therefore, these structural parameters indicate that the aggregates have characteristic dimensions in the range of tens of nanometres, much bigger than the complex previously reported by Vulliez Le Normand et al. [15]. The linear PvRON2 (2035–2074) peptide was very difficult to solubilize both in aqueous solution and in organic solution. This suggested that these aggregates correspond mainly to the linear PvRON2 (2035–2074) peptide.

## Conclusions

The structures of peptides (cyclic and linear) based on PvRON2 show a predominance of PPII conformation in aqueous solution. According to the results obtained in this work, the disulfide bridge between C2051 and C2063 cause a great conformational restriction to the cyclic peptide. This conformational restriction, nonexistent in the linear peptide could help the cyclic peptide to adopt the correct conformation to interact with PvAMA1 showed in the work of Vulliez Le Normand et al. [15].

Peptide and recombinant protein with an S–S bond between C2051 and C2063 were able to interact with PvAMA1-His_6_. The C-to-A and C-to-S (that is chemically similar to C) changes in the recombinant protein and linear peptide respectively, don’t show interaction with PvAMA1, proving that the S–S bond between C2051 and C2063 is crucial for the interaction between PvRON2 and PvAMA1.

These results together with the results obtained by Vulliez Le Normand [15], Bermudez et al. [16], and Srinivasan et al. [20, 21, 34] strongly suggest that PvRON2 and complex PvAMA1/PvRON2 can be developed as therapeutic alternatives to prevent the parasite invasion of RBCs that occurs in malaria or as antigens for vaccines.

## Additional file

**Additional file 1.** Peptide production.
