# Supplementary material for: Structure of Rhoptry Neck Protein 2 is essential for the interaction in vitro with Apical Membrane Antigen 1 in Plasmodium vivax
Source: Malar J. 2019 Jan 25;18:25. doi: 10.1186/s12936-019-2649-6 (PMC6347818; doi:10.1186/s12936-019-2649-6)
Supplement: Supplementary file 1 — Additional file 1. Peptide production. [file 12936_2019_2649_MOESM1_ESM.docx]

*Amino acid composition of PvRON2 (2035-2074) cyclic:* A 10.3% (4), D 5.1% (2), C 5.1% (2), Q 10.3% (4), G 5.1% (2), H 2.6% (1), I 10.3% (4), L 2.6% (1), K 5.1% (2), P 10.3% (4), S 7.7% (3), T 15.4% (6), Y 2.6% (1), V 5.1% (2), X (Nle) 2.6% (1). *ESI Mass:* PvRON2 (2035-2074) cyclic (MW Theoretical: 4053,6287) obtained m/z; 4053,63.

*Amino acid composition of PvRON2 (2035-2074) linear:* A 10.3% (4), D 5.1% (2), Q 10.3% (4), G 5.1% (2), H 2.6% (1), I 10.3% (4), L 2.6% (1), K 5.1% (2), P 10.3% (4), S 12.8% (5), T 15.4% (6), Y 2.6% (1), V 5.1% (2), X (Nle) 2.6% (1).

*ESI Mass:* PvRON2 (2035-2074) linear (MW Theoretical: 4023,5114) obtained m/z; 4023,54.

**Results of synthesis and purification of** **PvRON2 (2035-2074) based peptides fragments**

The PvRON2 (2035-2074) linear peptide was purchased from Peptide 2.0 (Chantilly, VA) with a level of purity higher 95%.

The synthesis of PvRON2 (2035-2074) cyclic (Figure 1S) was performed by the standard Fmoc chemistry using DIC-HObt as couplings agents in DCM/DMF (1:1). The yield of the PvRON2 (2035-2074) cyclic synthesis, using the aforementioned protocol were 1670 mg.

**Figure S1: Peptides sequence localization in *Pv*RON2 protein.** *Pv*RON2 (2035-2073) (red) represent peptides based in *Pv*RON2. SP, signal peptide. TMD, putative transmembrane domain. *Pv*RON2 scheme based in models of Tang *et al*, 2012, and Vulliez-Le Normand *et al*, 2012 [1,2]. In both peptides, M was changed by Norleucine (Nle) (X) to avoid the oxidation process of methionine in peptide synthesis [3]. In linear peptides, C was changed by S.

The LC retention times of PvRON2 (2035-2074) cyclic in C18 analytical column were 13.62 min (before cyclization), and 22.18 min (after cyclization)**.** Combination of two of protocols tested has better results, and 8.0 mg of homogeneous material was recovered from all purification procedures for cyclic peptide, corresponding to a purification yield of 0,48%. The purified PvRON2 (2035-2074) cyclic sequence gave the expected amino acid composition and molecular weight (m/z 4053.6287 by electrospray MS). Figure 2S shows the analytical HPLC profile of the purified PvRON2 (2035-2074) cyclic. The purity obtained was greater than 95% (figure 2SC). Our results indicated that was possible to synthesize PvRON2 (2035-2074) cyclic with high degree of purity.


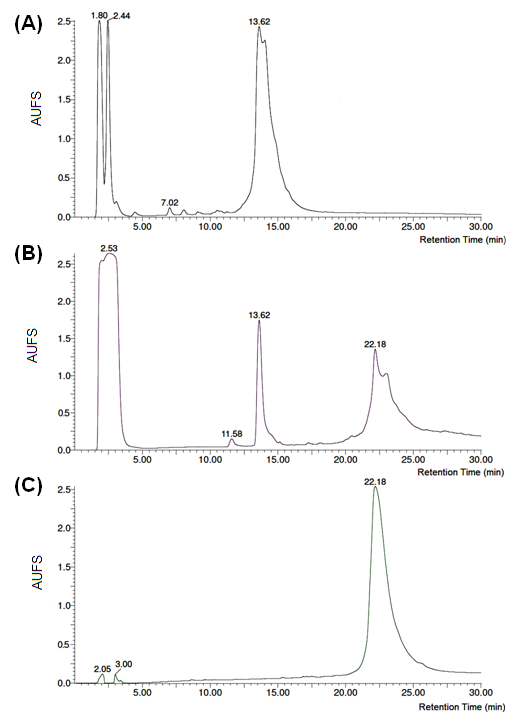


**Figure S2:** HPLC profiles of peptide PvRON2 (2035-2073) cyclic obtained by Fmoc synthesis strategy. **(A)** Before cyclization (RT: 13.62 min), **(B)** the same product after cyclization (RT: 22.18 min), **(C)** the same product after purification (RT: 22.18 min).

1. Tang J, Dai Y, Zhang H, Culleton RL, Liu Y, Zhao S, et al. Positive diversifying selection on Plasmodium vivax RON2 protein. Parasitology [Internet]. 2012;139:709–15. Available from: http://www.ncbi.nlm.nih.gov/pubmed/22321319%5Cnhttp://journals.cambridge.org/action/displayAbstract?fromPage=online&aid=8547466

2. Vulliez-Le Normand B, Tonkin ML, Lamarque MH, Langer S, Hoos S, Roques M, et al. Structural and functional insights into the malaria parasite moving junction complex. PLoS Pathog. 2012;8.

3. Nagasundarapandian S, Merkel L, Budisa N, Govindan R, Ayyadurai N, Sriram S, et al. Engineering protein sequence composition for folding robustness renders efficient noncanonical amino acid incorporations. ChemBioChem. 2010;11:2521–4.
